# Near-term climate change impacts on sub-national malaria transmission

**DOI:** 10.1038/s41598-020-80432-9

**Published:** 2021-01-12

**Authors:** Jailos Lubinda, Ubydul Haque, Yaxin Bi, Busiku Hamainza, Adrian J. Moore

**Affiliations:** 1 School of Geography and Environmental Sciences, Ulster University, Coleraine, UK; 2 Department of Biostatistics and Epidemiology, University of North Texas Health Science Centre, Fort Worth, TX 76107 USA; 3 School of Computing, Ulster University, Newtownabbey, UK; 4 Ministry of Health, National Malaria Elimination Centre, Lusaka, Zambia; 5 Department of Geography, University of Florida, Gainesville, FL USA; 6 Emerging Pathogens Institute, University of Florida, Gainesville, FL USA

**Keywords:** Malaria, Environmental impact, Climate-change ecology

## Abstract

The role of climate change on global malaria is often highlighted in World Health Organisation reports. We modelled a Zambian socio-environmental dataset from 2000 to 2016, against malaria trends and investigated the relationship of near-term environmental change with malaria incidence using Bayesian spatio-temporal, and negative binomial mixed regression models. We introduced the diurnal temperature range (DTR) as an alternative environmental measure to the widely used mean temperature. We found substantial sub-national near-term variations and significant associations with malaria incidence-trends. Significant spatio-temporal shifts in DTR/environmental predictors influenced malaria incidence-rates, even in areas with declining trends. We highlight the impact of seasonally sensitive DTR, especially in the first two quarters of the year and demonstrate how substantial investment in intervention programmes is negatively impacted by near-term climate change, most notably since 2010. We argue for targeted seasonally-sensitive malaria chemoprevention programmes.

## Introduction

An estimated 3.4 billion people in 92 countries are at risk of malaria infection^1^. Malaria eradication is possible within a generation, although achieving this goal requires improvements and continuous progress in socioeconomic and environmental trends^1^. At the same time, there needs to be improved coverage of current malaria intervention activities. The World Health Organisation (WHO) indicates that climate change could cause approximately a quarter of a million additional deaths per year between 2030 and 2050, from malnutrition, malaria, diarrhoea and heat stress^2^. It has been noted that the impacts of climate change on malaria transmission are already being felt in most regions. However, some places continue to make good progress against malaria over the last decade^3^. Such variations persist across climate variables (e.g. temperature) and spatial scales down to the smallest level where the changes have a direct effect on individual wellbeing and survival. The general association of variable malaria incidence with a range of climate measures has been evident at various geographical scales from the global to the very small area level within countries^4–15^.

Zambia, for example, has experienced considerable progress in reducing malaria mortality in the last 2 decades^16,17^. This progress came as a result of progressively better case management, prompt diagnostics [e.g. using rapid diagnostic tests (RDTs)]^18^ and a large scale-up of malaria interventions through vector control measures such as insecticide-treated nets (ITNs) and indoor residual spraying (IRS)^17,19,20^. Many districts within the country have transitioned from having a ubiquitously high malaria mortality burden to having only a few deaths annually^21^. Previously, high rates were mainly attributed to delays in seeking treatment, self-medication, and low immunity, especially in children aged under 5 years old.

Between 2000 and 2016, Zambia's within-country^22^ malaria incidence rates generally declined in most areas before increasing again post 2008. This trend has occurred despite improvements in the quality and availability of RDTs since 2009, and the uniform distribution of interventions applied as a national strategy over the intervening period. Consequently, while Zambia experiences a moderate-to-high and spatially heterogeneous malaria transmission pattern countrywide^23^, the question remains as to why the burden of malaria has not decreased in all areas despite the application of various control measures^24^.

Climate change, among other factors, has been cited as a potential cause for the persisting malaria incidence and the notable increases in some areas^21,25^ as the condition is particularly sensitive to changes in temperature and rainfall. Depending on the levels of transmission, which are fundamentally driven by baseline environmental conditions, areas can respond differently to the introduction of intervention measures. For example, areas with high intense transmission will respond differently to the introduction of the same types and levels of interventions than those in low and moderate transmission regions^26^ given that climatic influences are a consequence of both change over time and the baseline climatic conditions.

The distribution of mosquito vectors also depends on a range of factors such as the biology of the mosquito species, the local ecology, and the effectiveness of vector control programmes^27,28^. Climatic factors are also strongly associated with mosquito reproduction habits, whereby extreme conditions can restrict their longevity resulting in potential changes in vector density and infections. Recognising this connection, studies of the impact of purportedly anthropogenic induced climate change on malaria have increased in recent years^15,25,29–34^.

In order to understand the role of short term changing environmental conditions (i.e. near-term climate change) in explaining different malaria trends at the sub-national district level in Zambia, we investigated the potential role of climate variables in transmission dynamics over a 17-year period (2000–2016). We selected all districts that showed a declining trend in malaria incidence and compared them with those that had an increasing trend with respect to the temporal trends in quarterly temperature (maximum, minimum, and diurnal ranges), precipitation, the normalised difference vegetation index (NDVI) and elevation.

## Methods

### Study area: demographics and information on malaria

Zambia is a Southern African country of 752,000 km^2^, with a population of c. 17 million people and has a tropical climate^35^. For this study, we acquired estimated district level populations from intercensal and postcensal exponential population growth models based on the Central Statistics Office (CSO) reports from 2000 and 2010. Routinely collected malaria epidemiological data were obtained from Zambia's Ministry of Health (MoH) through the National Malaria Elimination Centre (NMEC).

Since 2009, all confirmed malaria incidence data were derived from a laboratory diagnostic test or a rapid diagnostic test (RDT) result, while the presence of malaria symptoms, including a fever but with no confirmed diagnostic testing, was defined as unconfirmed (or clinical) malaria. We could not differentiate between clinical and confirmed malaria pre-2009 as the data was not separated in its original reporting. Both cases diagnosed using clinical symptoms (unconfirmed) and those confirmed using RDT/Microscopy were combined when reporting and are therefore not separable. Post-2008, the data is reported separately, and we used both confirmed, as well as positivity rate adjusted unconfirmed cases.

The data were also adjusted for reporting completeness, missingness, treatment-seeking, and outliers at the district level. Completeness of reporting was calculated, as shown in the equation below. When no values were available, report completeness was assumed to be 100% so that the most conventionally high disease burden estimate was returned as described in^36^ and Equation below.

$${\text{Completeness }} = \frac{{\text{Total reports recieved}}}{{\text{Total reports expected}}}$$

Total reports expected is the total number of monthly reports that should be received in a given reporting year. For district level reporting, this is multiplied by the number of health facilities in the district. So, if a district has 10 health facilities, we would expect 10 health facilities × 12 months = 120 reports.

With regards to spatial bias in data completeness, we tested health facility-level data, for rural–urban clustering using the Getis–Ord Gi* Spatial Statistic and found that reporting completeness was random with a z-score = 0.048786 and *p* value = 0.96. Further data adjustments involved first examining individual district counts, before testing for the presence of outliers or the presence of spurious values using Cooks distance test. The data's completeness reporting during the period of study was calculated at district-level. Missingness was dealt with at the district level and stood at 0.1% among reported cases. We used multiple imputations to create several complete versions of the dataset and replaced missing values with plausible data values^37^ using the MissForest R package^38^. Random Forest was trained on the observed values from a matrix to estimate the missing values and impute the 0.1% of missing records in the data.

The overall adjustment was made using the equation:

$$= {{\left( {\frac{{Cases_{public\;confirmed} + (Cases_{public\;presumed} \times Test\;positivity\;rate_{public} )}}{Reporting\;completeness}} \right)} \mathord{\left/ {\vphantom {{\left( {\frac{{Cases_{public\;confirmed} + (Cases_{public\;presumed} \times Test\;positivity\;rate_{public} )}}{Reporting\;completeness}} \right)} {Treatment\;seeking\;rate}}} \right. \kern-\nulldelimiterspace} {Treatment\;seeking\;rate}}$$

All malaria data was aggregated into quarterly time periods as this was the only temporal resolution at which data was available between 2000 and 2008. Thereafter data was available monthly but was aggregated into quarters in line with that of pre-2008. The quarterly reporting periods closely align with malaria transmission seasonality in the country. All vector control data (ITNs and IRS) were obtained from the MoH at the district level and were consistently measured and reported throughout the study period.

### Climate and ecological data

Environmental variables were obtained from satellite-based imagery datasets. Daily precipitation data were extracted from the Climate Hazards Group archive with a spatial resolution of 5 × 5 km^39^; daily temperature data were sourced from NCEP Climate Forecast System Reanalysis (CFSR) at the 20 × 20 km level^40^, and Normalised Vegetation Index (NDVI) was obtained from Copernicus Global Land Service (CGLS) at the 1 × 1 km and 10-day spatio-temporal resolutions^41,42^. All the environmental variables were extracted by district using the R Program raster package^43^.

We extracted aggregated quarterly mean, minimum and maximum seasonal rainfall (mm) averages as well as mean (Tmean), maximum (Tmax), and minimum (Tmin) values of temperature (°C) for the period from January 2000 to December 2016 for all 72 districts. Our choice of the two primary climate variables (temperature and rainfall) was based on current evidence from the literature confirming an existing relationship between malaria, temperature and rainfall^24,44–51^. Seasonality was matched with yearly quarters calculated as January-March (Quarter 1), April–June (Quarter 2), July–September (Quarter 3), and October–December (Quarter 4). Most published studies show a 1–3 months lag in incidence reporting^4,52–55^, which fitted with our quarterly definition. We applied computations of mean seasonal (quarterly) trend detection and change-point analysis for Tmax, Tmin, Tmean, mean rainfall, and maximum rainfall variables, to detect any trending of climate change points in the data. Diurnal temperature range (DTR) was computed and extracted from the daily Tmin and Tmax variables for the duration of the study.

### Data analysis and overall models used

All the variables included were tested for association and collinearity, as shown in Supplementary Table S1. Collinearity between Tmin and Tmax was a contributing factor in the decision not to model mean temperature (Tmean) together with Tmin, Tmax or DTR. We did, however, model it separately for comparison purposes. Variables included were tested for association using univariate statistics, and collinearity tested using variance inflation factors (VIF). All variables with high VIFs, including relative humidity which was highly collinear with temperature and rainfall, were excluded. Summaries of these pre-analysis tests are shown in Supplementary Table S1.

We implemented mixed-effects models using binomial regression analysis to establish the independent effects of environmental conditions on the malaria incidence trends exhibited by each district. An example of the simplified equation follows:

1 $${\text{E}}\left[ {\text{y}} \right] = \exp \left( {{\text{X}}\upbeta + {\text{Zu}} + {\text{e}}} \right)$$

where Z is a design matrix similar to X, and u represents a vector of parameters like β^56^. The complex form is as in Eq. (2)^57^: where Y is a dependent variable; β_0_ is the intercept; and β_1_, …, β_x_ represent the slope of predictors X_1_,…, X_x_ respectively. Meanwhile, u_0,_ …, u_x_ are the level-2 residuals; ${\upgamma }_{0},\dots , {\upgamma }_{\mathrm{x}}$ are the means of ${\upbeta }_{0,}\dots ,{\upbeta }_{\mathrm{x}}$ and ${\mathrm{u}}_{0},\dots , {\mathrm{u}}_{\mathrm{x}}$ are levels of residuals contributing to $\mathrm{r}$ which denotes level-1 residuals respectively.

$${\text{Y}} = \upbeta _{0} + \upbeta _{1} {\text{X}}_{1} + \upbeta _{2} {\text{X}}_{2} + \upbeta _{3} {\text{X}}_{3} \cdots + {\text{r}}$$

r follows a univariate normal distribution $\mathrm{r}\sim \mathrm{N}(0,\upepsilon ),$ and ${\mathrm{u}}_{0},\dots {\mathrm{u}}_{\mathrm{x}}$. $\mathrm{u}=[{\mathrm{u}}_{0},\dots {\mathrm{u}}_{\mathrm{x}}]\mathrm{T}$, and follows a multivariate normal distribution $\mathrm{u}\sim \mathrm{MN}\left(0,\Sigma \right).$ Explanatory variables included interventions and climatic variables, while district was included to capture random effects.

We then ran an analysis on Zambia's malaria trends between 2000 and 2016 first by classifying temporally varying random effects of each district's spatio-temporal trends into declining, increasing, or constant^58^. We used a Bayesian hierarchical random mixture model implemented with an inference through a Metropolis-coupled Markov chain Monte Carlo (MCMCMC) model. The inference was based on a sample size of 200,000 iterations, *M* = 4 parallel chains, a thinning of the degree of 10, and a burn-in of 20,000. We used Gelman's trace plots and visual diagnostics to determine the convergence of the models^59,60^.

The general model structure and formulae of the temporal model are given by the equation :

$$Y_{kt} \sim p\left( {y_{kt} {|}\mu_{kt} } \right), \quad {\text{where}}\;K = 1, \ldots ,K,\;t = 1, \ldots ,N,$$

2 $$g\left( {\mu_{kt} } \right) = O_{kt} + {\varvec{X}}^{T}_{kt} {\varvec{\beta}} + \phi \mathop \sum \limits_{s = 1}^{s} \omega_{ks} f_{s} \left( {t{|}{\varvec{\gamma}}_{{\varvec{s}}} } \right)$$

In the equation, K denotes district (an administrative geography), t = time, while malaria trends *f*_*s*_*(t|γS)* as estimated in the study were represented by (a) Constant trend—*β*_*1*_; (b) Linear increasing trend—*β*_*1*_ + *γ*_*1*_*t*, with *γ*_*1*_ > 0; and (c) Linear decreasing trend—*β*_*1*_ + *γ*_*2*_*t,* with *γ*_*2*_ < *0*. The trends classification is summarised according to the following:

- Constant: $$f(t) = 0$$.
- Linear $: f(t|\gamma ) = \gamma t$, which can be constrained as increasing via the prior specification by $\gamma \sim N(0, 1000){\mathbb{I}}[\gamma > 0]$ or decreasing via $\gamma \sim N(0, 1000){\mathbb{I}}[\gamma < 0]$, whereby ${\mathbb{I}}[.]$ is an indicator function.

A more detailed description of this model is given elsewhere^58,61^.

The model outputs were used to map the malaria trends of the 72 districts over 17 years of the study period, from which the areas that exhibited an increasing trend or declining trend in malaria incidence risk among both under 5 children and those 5 years and older were selected. We ran regression against environmental and intervention variables known to have a biologically plausible effect that either stifles or exacerbates malaria transmission. These included climate variables such as temperature, rainfall, normalised difference vegetation index (NDVI), all known to affect mosquito vectors, and malaria indoor residual spraying (IRS) and insecticide-treated nets (ITN), known interventions as vector prevention or management mechanisms.

Our preliminary analysis explored the regression suitability of fixed and random effects models for the variables. The tests used are presented in Supplementary Table S1. The diagnostic plots obtained from both linear models informed decisions made from our pre-analysis comparisons and random-mixed models diagnostics using plots from generalised linear and logistic regression models (Supplementary Fig. S1). To detect trends in climatic variables, we utilised several climate-sensitive tests such as linear regression, and other parametric and non-parametric statistics as applied in other studies^62,63^. We detected distribution trends at 95% significance by the Mann–Kendall test, Multivariate (multisite) Mann–Kendall test, Pettit’s test, and Seasonal slope estimator. The Cox-Stuart Trend Test and the Buishand's Range Tests helped in change point detection and homogeneity testing in climatic variables.

The spatiotemporal mixed model allowed for spatio-temporal autocorrelation via random effects, which capture autocorrelation remnants in the malaria data after the impact of the known covariates have been accounted for. We also tested for the presence of spatial autocorrelation in the data by computing the residuals from a simple over-dispersed Poisson log-linear model that incorporated the covariate effects.

## Results

### Malaria incidence trends from 2000 to 2016

Zambia's malaria incidence showed a strong spatial shift between 2000 and 2016, mainly from a few spontaneous higher transmission districts, with high endemicity in the southern and south-western regions to a predominantly northern and north-eastern region of the country. A significant difference was observed in the spatial and temporal patterns of malaria over the study period. Similarly, several agriculture based studies^64–68^ are consistent with the observed shifts in temperature and rainfall, showing declining rain but increasing temperature trends in southern most areas but the opposite for northern and north-eastern parts of Zambia.

**Figure 1:**
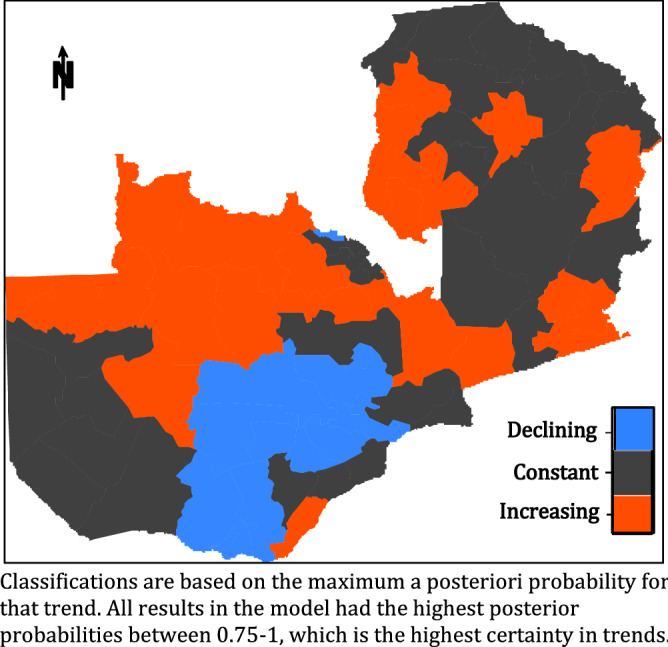
Malaria trends in Zambia districts between 2000 and 2016. Classifications are based on the maximum a posteriori probability for that trend.All results in the model had the highest posterior probabilities between 0.75 and 1, which is the highest certainty in trends.

Figure 1 shows the posterior probabilities of disease trends assigned to each district, categorised as either having an increasing trend, a constant trend or a decreasing trend. The classification is based on the maximum posterior probabilities to capture uncertainty—the darker/deeper the shading, the higher the posterior probability for that trend and vice versa. There was very little posterior uncertainty in the trend classifications for all districts. Of Zambia’s 72 districts, 25 (35%) were identified with increasing malaria. In contrast, 13 (18%) were classified with declining malaria, and 34 (47%) had neither declining nor increasing malaria (i.e. a generally mixed non-significant trend for the two population age categories). There is a very distinctive spatial pattern of district clustering with areas of declining malaria mostly being located in the southern part of the country.

**Figure 2:**
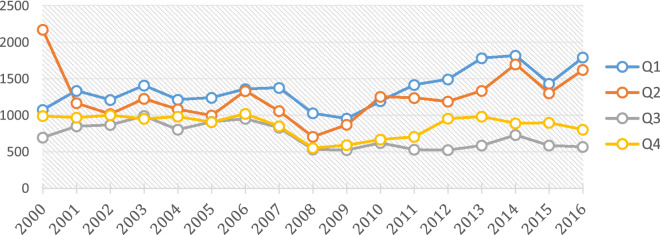
Mean seasonal/quarterly malaria transmission 2000–2016.

During the study period, there was a uniform seasonal malaria trend between 2000 and 2008 (Fig. 2). After 2008, the first and second quarters (Q1 and Q2) exhibit a general increase in the mean incidence per 1000 population, Q3 remained relatively constant (pre and post-2008) while Q4 maintained the new lower level attained by 2008. The figure shows that most of the observed increases in seasonal malaria during the study period were due to changes in Q1 and Q2, representing the months from January to June. However, this trend was not consistent across all 72 districts. This is particularly interesting in the post-2008 Q3 and Q4 off-season quartiles in areas with either a constant or increasing trend (Fig. 4b,c) where we might have expected the numbers to be significantly decreasing due to non-malaria case removals as observed in the areas with decreasing trends (Fig. 4a), but the trend is actually slightly increasing. The same general trends (Supplementary Fig. S11) are exhibited spatially and temporally across the country in the various malaria indicator surveys from 2006–2015^69–73^.

### Short-term climate variable trends in areas classified with declining or increasing malaria

**Figure 3:**
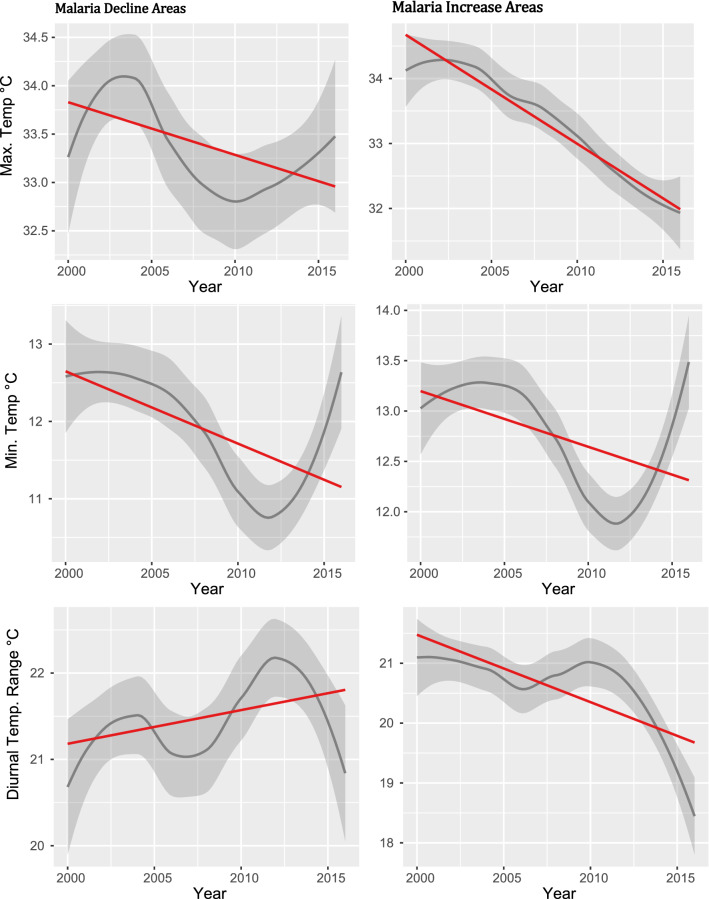
Temporal trends of temperature variables (using LOWESS smoothing function). Grey represents confidence intervals (95% CI) with 75% data. The red-line represents a linear best fit of the distribution.

The results in Fig. 3 show that the temporal trend for temperature was generally declining in areas with declining malaria. There was a very small but significant decline in Tmax with slope = − 0.05, R^2^ = 0.005 (95%, *p* = 0.03) and equally small but significant increase in Tmin with slope = 0.09, R^2^ = 0.02 (95%, *p* = 0.001). This supports the observed non-significant increase (slope = 0.04, R^2^ = 0.002, *p* = 0.12) in the diurnal range and indicates that temperature has been reasonably stable in areas of declining malaria. The DTR also has a strong negative relationship with malaria. Breaking the analysis visually into three temporal segments (2000–2005; 2006–2010; and 2011–2016) indicated a substantial relationship between climate variables and malaria in the first two time periods but a slightly weaker relationship in the latter. The results from the segmented analysis did not add any additional information to the content presented here.

The temperatures in areas with increasing malaria trends also declined. However, there were much greater significant declines in both Tmax and Tmin (slopes = − 0.14, and − 0.07; R^2^ = 0.04 and 0.01, *p* < 0.05), respectively. There was also a significant difference in the two slopes as the Tmax had a slope twice as that of Tmin (slope, *p* = 0.004), thus validating the observed significant decline in DTR (95% CI) during the study period.

Tables 1 and 2, and Supplementary Figs. S2 to S4 show further details of the regression model results of environmental variables against malaria incidence. In areas of declining malaria, only Tmax and DTR had significant negative correlations while Tmin had a positive effect. NDVI, elevation, and rainfall (min and max) were not significant (see Supplementary Figs. S2 and S3). For those areas with an increasing malaria trend, mean rainfall and temperature (Tmin, Tmax, and DTR) showed significant effects. In contrast, maximum rainfall and elevation had no significant relationship with malaria (Supplementary Fig. S2 and S4). Overall, the results demonstrate a much stronger correlation of environmental variables with malaria in areas of declining malaria.

**Table 1:** Regression model of environmental variables and malaria.

Variable	Estimate	Std. error	Pr( > |z|)
**Areas with declining malaria (AIC = 34,647)**			
Diurnal range	− 0.19859	0.07828	0.0112*
Tmin	0.16239	0.07131	0.0228*
Tmax	− 0.22441	0.08876	0.0115*
Elevation	− 0.06927	0.09595	0.4704
NDVI	0.08429	0.07011	0.2293
Mean rain	− 0.01751	0.05218	0.7372
Max rain	− 0.06918	0.04707	0.1417
**Areas with increasing malaria (AIC = 7842)**			
Diurnal range	− 0.08990	0.01617	2.71e−08***
Tmin	0.028166	0.013510	0.0371*
Tmax	− 0.122918	0.016230	3.64e−14***
Elevation	− 0.04081	0.05617	0.4675
NDVI	− 0.04081	0.01687	< 2e−16***
Mean rain	− 0.04382	0.01702	0.0100*
Max rain	− 0.00831	0.01209	0.4919

Signif. codes: 0 ‘***’ 0.001 ‘**’ 0.01 ‘*’ 0.05 ‘.’ 0.1 ‘ ’ 1.

**Table 2:** Summary of DTR seasonality trends vs malaria seasonality.

Season	Slope	Intercept	R^2^	F	*p*
**Temperature (DTR)—areas of malaria decline**					
Q1	− 0.10892	235.7	0.13	2.33	0.15
Q2	− 0.04198	105.7	0.02	0.33	0.57
Q3	0.117428	− 210.4	0.30	6.52	0.02*
Q4	0.1897	− 358.7	0.37	8.96	0.009**
**Seasonal malaria trend—areas of malaria decline**					
Q1	− 83.7099	168,746.7	0.79	57.97	1.58E−06****
Q2	− 99.2363	199,932.3	0.70	34.78	2.93E−05****
Q3	− 64.8745	130,681.8	0.80	58.90	1.43E−06****
Q4	− 66.9569	134,865.9	0.86	95.30	6.85E−08****
**Temperature (DTR)—areas of malaria increase**					
Q1	− 0.23652	491.3	0.67	30.63	5.72E−05****
Q2	− 0.16497	351.5	0.43	11.23	0.004381**
Q3	− 0.00911	42.9	0.002	0.03	0.865916
Q4	− 0.03933	100.1	0.035	0.54	0.475054
**Seasonal malaria trend—areas of malaria increase**					
Q1	99.36241	− 197,667	0.75	43.96	8.01E−06****
Q2	69.81311	− 138,490	0.41	10.64	0.005**
Q3	8.418798	− 15,953.9	0.096	1.60	0.23
Q4	22.35414	− 43,681.6	0.23	4.37	0.054

Further analysis to examine the more recent trend from 2010 to 2016 revealed an overall decline of DTR across the whole country (Supplementary Fig. S5). This is validated by specific trend-based results, which show that districts with increasing malaria had increases in Tmin (slope = 0.19, R^2^ = 0.02), but a continued decline of Tmax (slope = − 0.22, R^2^ = 0.01). Both trends (statistically significant - 95%), further denote a continuous decline in DTR with higher regression coefficients during the post-2010 period. In comparison, areas with declining malaria experienced a significant (*p* < 0.05) increasing trend in Tmin (slope = 0.22, R^2^ = 0.02) but a non-significant (*p* > 0.05) increasing trend in Tmax (slope = − 0.08, R^2^ = 0.001). There was no significant difference in the slopes of Tmin and Tmax and the trend for DTR, which, although declining, was not statistically significant.

A direct comparison between the areas with increasing malaria and those with declining malaria showed that mean environmental conditions are currently more favourable in those areas with increasing malaria than in those areas with declining malaria (Supplementary Table S2). A hypothesis test of differences in environmental variables using Welch's t statistic for unequal variances returned a statistically significant result in the differences between paired groups of Tmean, Tmin, DTR, Mean daily rain, and Max daily rain, with only Tmax non-significant (*p* value = 0.69).

Supplementary Fig. S6a and b show that the standard deviations of random effects relative to the model outcomes between districts with increasing malaria and those with declining malaria are very different. Declining areas tend to have a more uniformly low standard deviation about the intercept (Supplementary Fig. S6a), with random effects quantiles ranging between − 0.05 and 0.05 (See Supplementary Fig. S7). In contrast, the large variations existing among districts with increasing malaria, indicate that there may be different probabilities of success depending on the interaction in response to model variables with wider random effect quantiles at least seven times higher (range between − 0.37 and 0.37) than those of districts with declining malaria) (see Supplementary Figs. S6b and S8).

### Seasonality trends

**Figure 4:**
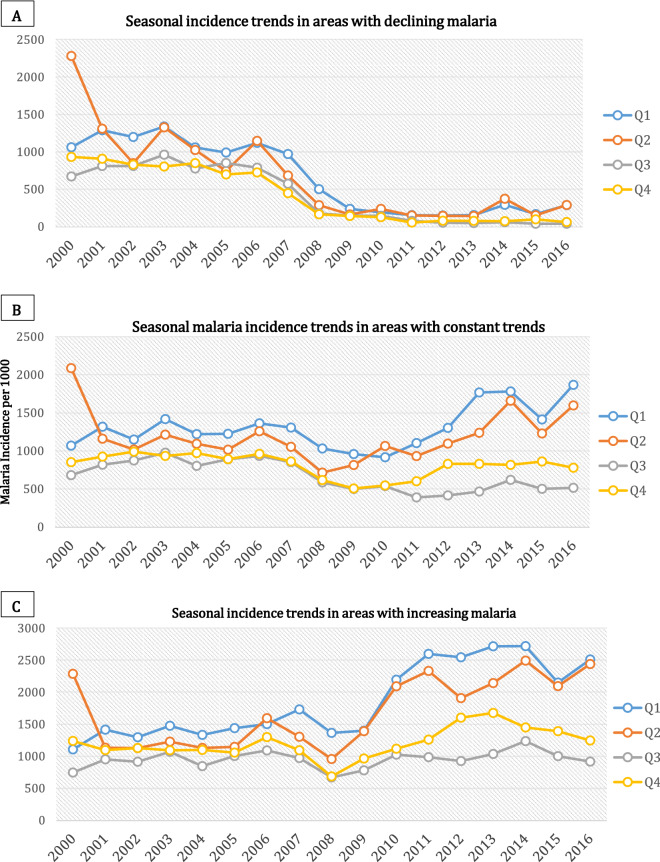
Seasonality of malaria in areas of decline, increase, or constant trends.

Further analysis of seasonal malaria between areas with differing trends (increasing, decreasing or constant) indicated a direct relationship with variances in the seasonal DTR. For example, there was no spatial or temporal seasonal difference in areas with declining malaria, with all seasons experiencing similar declining trends (95%) across the study period. The same was true for seasonal DTR, which exhibited a non-significant declining trend in Q1 and Q2, but significant increasing trends in Q3 and Q4 (Fig. 4a and supplementary S9a).

In contrast, areas with increasing malaria had distinguishable significant increases (*p* < 0.05) in Q1 and Q2, which become more acute after 2008 (Fig. 4c). A possible argument that the observed differences might be an artefact of changes in reporting is questionable, as we would expect that improved reporting should have resulted in increased trends across all the annual seasons and all districts. Figure 4c, for instance, shows a clear split in trends between the first half of the year and the second half with a significant increase (*p* < 0.05) in the first two quarters. The opposite was true for DTR (supplementary Fig. S9c) which had significant declines (*p* < 0.05) in Q1 and Q2, but a declining trend in Q3 and Q4 which was not statistically significant (see Table 2 for full details). Figures 4b and Supplementary Fig. S9b characterise the trends presented above as falling mostly within non-significant trends in either malaria or temperature variables and are not discussed here.

### Trends in malaria vector interventions

We investigated the role of malaria interventions, particularly mosquito nets (ITNs/LLINs) and indoor residual spraying (IRS) (Supplementary Fig. S10). The results indicate that there was no significant difference in intervention distribution and coverage reported between the two areas (slope = 0.26, *p* > 0.05). The regression statistics also indicate that the slopes of LLIN coverage are not significantly different (95%) from zero, nor are the intercepts of the two trend areas. IRS, however, showed that there was a significant difference in the amount of spraying between the two areas. In the regression analysis between malaria and intervention variables (LLINs and IRS) (see Supplementary Fig. S2), we found that LLINs and IRS showed negative effects in areas of declining malaria. However, the IRS was not statistically significant, while neither showed any significant effects in areas with increasing malaria. For comparability purposes, ITN calculations were based on the recommended WHO calculation accounting for a 30% attrition rate^74–76^. Nonetheless, the comparison of effects between the two interventions should, however, be made with caution due to inherent differences in both the implementation time scales and duration of effectiveness (especially for IRS whose effectiveness also depends on the chemical half-life as well as the killing effect)^77^.

## Discussion

The results presented above confirm that there are prevailing spatio-temporal differences in malaria progress within Zambia over the period 2000–2016. Our analyses show that, while some areas exhibit continuous declines, others have experienced increasing trends, and some had no discernible change. These differences occur despite the reported uniformity in the deployment of interventions across the country.

Findings obtained from this study suggest that these stable changes in the seasonality of malaria incidence in districts where malaria is increasing (especially in Q1 and Q2) support the case for more targeted^78–81^ interventions, including seasonal malaria chemoprevention (SMC), which is individually administered. SMCs (like IPTi and IPTp) could effectively complement ongoing malaria control activities including LLINs, IRS, prompt diagnosis of suspected malaria, and treatment of confirmed cases with ACTs^82^. Such micro spatio-temporal targeting has the potential to be a more cost-effective means of reducing infections in those areas of highest risk to levels where they could become areas for potential elimination. Many studies undertaken in other countries^83^ have shown SMC to be effective to date (including *Burkina Faso, Chad, Gambia,* *Ghana,* *Guinea, Guinea Bissau, Mali, Niger, Nigeria, Senegal and Togo),* with up to 75% incidence reduction^82,84–90^ after being successfully introduced.

The results here demonstrate that there are significant near term spatio-temporal variations in environmental variables at the intra-regional district level in Zambia and that they are associated with similar variances in malaria incidence.

While the frequency of extreme weather events is typically used to measure climate change effects (i.e. extreme temperatures in minimums, maximums and range), the observed general temperature dynamics during the period of our analysis may imply that in some cases a narrowing of the temperature range could support more favourable all-year-round malaria transmission conditions compared to wider-ranges that may provide temporary transmission cut-offs (via extreme highs and lows). This could explain why malaria is consistently high in areas with a narrowing diurnal range, as shown in our study, where the narrowing is a consequence of near-term trends away from high and low-temperature extremes.

We have shown here that the change in malaria incidence rates correspond with significant increases in minimum temperature and declines in maximum temperature. This confirms the significance of the relationship between temperature and malaria, whereby a rise in minimum temperature causes a subsequent rise in malaria, as does a decline in maximum temperature. Some studies have tended to use the mean value of environmental variables to look for such effects. We show here that using mean values alone may not detect more subtle trends, like a narrowing of the diurnal temperature range, that produce more favourable transmission conditions and associated increases in malaria infection rates^91,92^. In fact, the findings here raise the question of the possibility that the positive effects of malaria interventions are being countered and diluted by the negative effects of changing climatic/environmental conditions that favour the proliferation of malaria (which the authors discuss more fully elsewhere^93^).

The observed increase in malaria incidence when the temperature in the malaria transmission suitability range narrows is consistent with theories which state that infectious rates are lower in periods of extremely high or low temperatures. This observation is corroborated by the argument that even minimal changes in temperature trends significantly increase parasite transmission because organisms can amplify transmission with such small variances^94^.

The use of DTR has the potential to be an additional, complimentary, if not alternative, measure that can be used to better understand the dynamics of the transmission range of malaria in spatio-temporal studies at the sub-regional level within all countries at risk of malaria infection.

Our results also support the contention^95^ that An. *gambiae* mosquitoes (which are one of Zambia's primary vectors) can experience substantial reduction effects in their vectorial capacity by over 80% with increasing optimum temperatures. Similarly, a decrease around the optimum temperature could increase transmission potential by over 600%. In contrast, increases in diurnal temperature range alone can reduce vectorial capacity by half, with range increases of around 9 °C or higher exacerbating the adverse effects on daily mosquito survival^95,96^.

Similarly, the climate in association with intervention coverage was reported to have contributed to the observed malaria reduction and resurgence in Zambia's children aged under 5 years^25^. Temperature and rainfall both influenced the potential for increased transmission intensity as determined by intra-annual climatic variability. A study of climate-related effects on malaria compared to those of interventions in 10 different countries showed that there was unintended over or underestimation of effects from interventions depending on the climate conditions in the baseline year^97^. The study reported that 30% of the countries had potentially under-estimated, another 30% had overestimated, and only 40% may have correctly attributed the effects of malaria interventions, depending on the climatic conditions prevailing in the baseline year. Our study results support the findings of these studies and others showing intra-country variations in climatic effects, such as rainfall and maximum temperature, on malaria incidence^98^.

It has been argued, quite correctly, that in order to impact improvements made in reducing malaria prevalence within countries, the potential negative effects of climate change would have to exceed the combined beneficial effects of economic development and increasing malaria control efforts^7^. We propose here that, based on the evidence since 2000, the true potential and positive effects of economic development and/or interventions in some parts of Zambia are being impacted and offset by the negative effects of near-term climatic change at the sub-regional district level.

Such a phenomenon has been observed elsewhere where, for example, the application of intervention programmes has been consistent throughout the year while malaria outbreaks tend to be seasonally high^99^. It may well be that while the observed temporal trends in temperature variance coincide with a significant up-scaling in national intervention programmes, the observed variations in vector response to these interventions and malaria infections (via insecticide-associated selection) may be largely controlled by local vector compositions^100^. It is worth noting, however, that the observed results on the effects of IRS and ITNs in areas with increasing malaria (Fig. S4) signify they have a lesser effect than they do in areas where malaria is declining. While it would have been expected that the more ITN and IRS are implemented, the rates of malaria would decline, the relationship identified here suggests that this is the case in areas with a declining trend but, despite an increase in interventions in areas with an increasing trend, malaria rates actually kept rising. This suggests that environmental factors are becoming more influential on the potential effects of interventions along with possible vector resistance to the chemicals used in some interventions.

Given the limited availability of information on mosquito resistance both spatially and temporally, it was not possible to investigate this in more depth. However, it should be noted that some of the observed increasing trends may be driven by existing (but unknown and unquantified) mosquito resistance as observed in recent studies that showed some mosquito resistance at a number of sentinel sites based on data collected between 2015 and 2018^22,101,102^. It has been estimated that resistance translates into a mean mosquito mortality change of  <0.15 between 2005 and 2017^103^.

Differing levels of urbanisation and rurality within and between districts may be another potential factor that influences IRS effectiveness, as urban districts have a higher probability of receiving IRS than their rural counterparts. This may be due to factors such as population density, ease of access and better-targeted structure surface suitability for spraying^104^, which potentially create a systematic bias favouring urban areas. Nevertheless, IRS remains a supplementary intervention strategy to LLINs. Where effective, it should reduce the annual seasonal peaks of malaria transmission equally, which is in contrast to the observed seasonal increases we found here. Similar results showing persisting malaria burden despite a scale-up of control interventions have been reported elsewhere^105^.

Therefore, while economic development and/or urbanisation may well be important in the fight against malaria in Africa, our results here indicate that the level of positive influence such factors have may well be negatively impacted and offset by intra-annual and inter-annual seasonality changes in environmental characteristics seasonally driven by near-term (and by inference part of long-term) climate change at sub-national spatial scales. We acknowledge that environmental conditions alone cannot sustainably control or eliminate malaria in the tropics, as their effects do not act in isolation. Nonetheless, our study has shown that changes in near-term small-scale environmental factors play a significant role in the complex matrix of factors that influence malaria rates. As such, these need to be incorporated as part of ongoing monitoring and analyses of rates and in elimination planning at the sub-national level. The relationship between intervention programmes and near-term environmental change may well be the difference between a successful malaria reduction/elimination program and persistent malaria transmission. Thus, if care is not taken, and climate change continues to drive these increases, there is a genuine danger that malaria in those areas of current decline might well start to increase again, thereby reducing the current malaria control and elimination agenda into a second failed global malaria program.

It is imperative to acknowledge that the impacts of short-term climate change on malaria are at hand and undeniable and that planning for adaptation, mitigation, and continuous monitoring is essential if we are to minimise the imminent effects, especially at the micro-scale community level. SMCs may provide an opportunity to target those areas with high seasonality impacts, especially in under 5 children. Consequently, it is essential that environmental change monitoring is considered alongside monitoring of interventions and prevalence rates so that appropriate preventive mechanisms to counteract transmission, such as SMC, can be introduced. Our study findings highlight how essential the discussion about intra-annual or inter-annual seasonality changes (in environmental characteristics) driven by near-term climate change and malaria still is today and demonstrates the seriousness of the potential consequences if it is ignored.

A potential limitation of this study is that the long time-series data used extends across some health policy changes, including the extensive introduction and use of RDTs since 2009. It is important to acknowledge that these changes may have partly influenced some of the observed trends.

## Supplementary Information

Supplementary Information.

## Data Availability

The data that support the findings of this study are available from the National Malaria Elimination Centre, but restrictions may apply to the availability of these data. Data are also available from the authors upon reasonable request and with the permission of the National Malaria Elimination Centre through the Ministry of Health.
