# Supplementary material for: Near-term climate change impacts on sub-national malaria transmission
Source: Sci Rep. 2021 Jan 12;11:751. doi: 10.1038/s41598-020-80432-9 (PMC7803742; doi:10.1038/s41598-020-80432-9)
Supplement: Supplementary file 1 — Supplementary Information. [file 41598_2020_80432_MOESM1_ESM.docx]

**Supplementary Figures and Tables for:**

**Near-term climate change impacts on sub-national malaria transmission**

Jailos Lubinda*^1^, Ubydul Haque^2^, Yaxin Bi^3^, Busiku Hamainza^4^, Adrian J. Moore^1^†

**Table S1**: **Summary of pre-analysis formal goodness-of-fit tests on the simulated residuals in GLMM**

| **Data structure** | **Test used** | **Result with** |
| --- | --- | --- |
| 1. **Outliers/Influential observations** | Cook’s distance | 149 observations |
| 1. **Multicollinearity** | Variance Inflation Factor (VIF) | Temperature (min, max, mean) |
| 1. **Zero-inflation** | DHARMa (test for zero-inflation) | None |
| 1. **Linear/non-linear structures** | Residual plots from linear models | Inconclusive presence of non-linear |
| 1. **Non-normality** | DHARMa (test for Uniformity) |  |
| 1. **Dispersion/ Heteroscedasticity** | DHARMa (test for dispersion) | Considerable presence of Over Dispersion |
| 1. **Temporal Autocorrelation** | Dubin Watson’s Test | Strong presence |
| 1. **Spatial Autocorrelation** | Moran’s I | Very weak presence |

Note: DHARMa R package was mainly used in testing from GLMM while the rest were mainly done for GLM or LM pre-analyses.

(1). For Uniformity test, we get too many residuals around 0 and 1 which means that we got too many residuals at the tails of the distribution than expected from the fitted models.

(2). Cook’s distance calculates observations with large values. In our data, 149 observations were deemed influential but these were too many to exclude.

(3). VIF was used to determine collinearity among explanatory variables denoted by very high VIF and correlation coefficients. Temperature variables (min, max, mean) values (≈200 000) and confirmed by high correlations (0.83 and 0.76) as well. We dropped the mean Temp variable based on biological sense and kept min and max.

(4). Although it seemed there was a weak presence of overdispersion at first, that disappeared once we corrected for dispersion

(6). Large overdispersion was found in lm, glm, and normal Poisson glmm models, hence the choice for negative binormial

(7). Spatial autocorrelation was not significant while temporal autocorrelation was present and significant.

**Figure S1: A summary of the diagnostic plots used to in the data analysis.**

Figure S1. Negative binomial glmmtmb: diagnostic plots showing corrected over dispersion and significant presence of spatial and temporal autocorrelation. These preliminary results helped us determine and confirm the right models for the data.


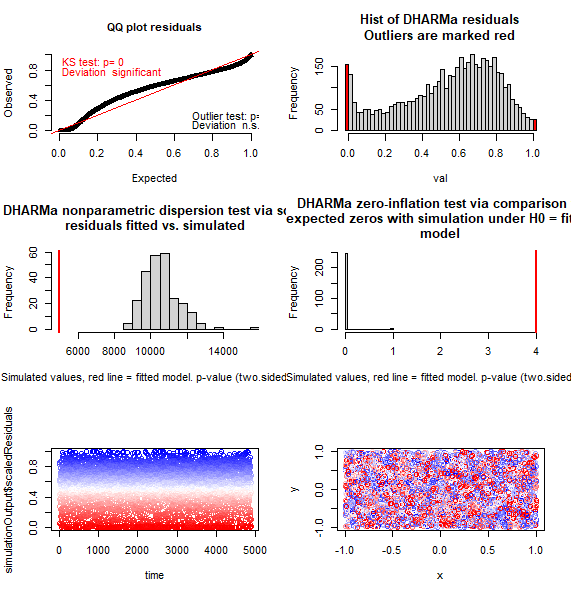


testSpatialAutocorrelation(simulationOutput) DHARMa Moran's I test for spatial autocorrelation data: simulationOutput observed = -0.00044970, expected = -0.00020433, sd = 0.00058953, p-value = 0.6773 alternative hypothesis: Spatial autocorrelation

testTemporalAutocorrelation(simulationOutput) Durbin-Watson test data: simulationOutput$scaledResiduals ~ 1 DW = 2.0259, p-value = 0.3648 alternative hypothesis: true autocorrelation is not 0

testZeroInflation(simulationOutput) DHARMa zero-inflation test via comparison to expected zeros with simulation under H0 = fitted model data: simulationOutput ratioObsSim = 500, p-value < 2.2e-16 alternative hypothesis: two.sided

testDispersion(simulationOutput) DHARMa nonparametric dispersion test via sd of residuals fitted vs. simulated data: simulationOutput ratioObsSim = 0.47051, p-value < 2.2e-16 alternative hypothesis: two.sided

testOutliers(simulationOutput) DHARMa outlier test based on exact binomial test data: simulationOutput outLow = 1.5400e+02, outHigh = 2.7000e+01, nobs = 4.8950e+03, freqH0 = 3.9841e-03, p-value = 0.1234 alternative hypothesis: two.sided

testUniformity(simulationOutput) One-sample Kolmogorov-Smirnov test data: simulationOutput$scaledResiduals D = 0.12669, p-value < 2.2e-16 alternative hypothesis: two-sided

**i)**

**ii)**

**iii)**

**iv)**

**v)**

**vi)**

**Figure S2: Comparative Visualizations of correlation coefficients**

Model1 = Areas of increasing malaria

Model2 = Areas of declining malaria


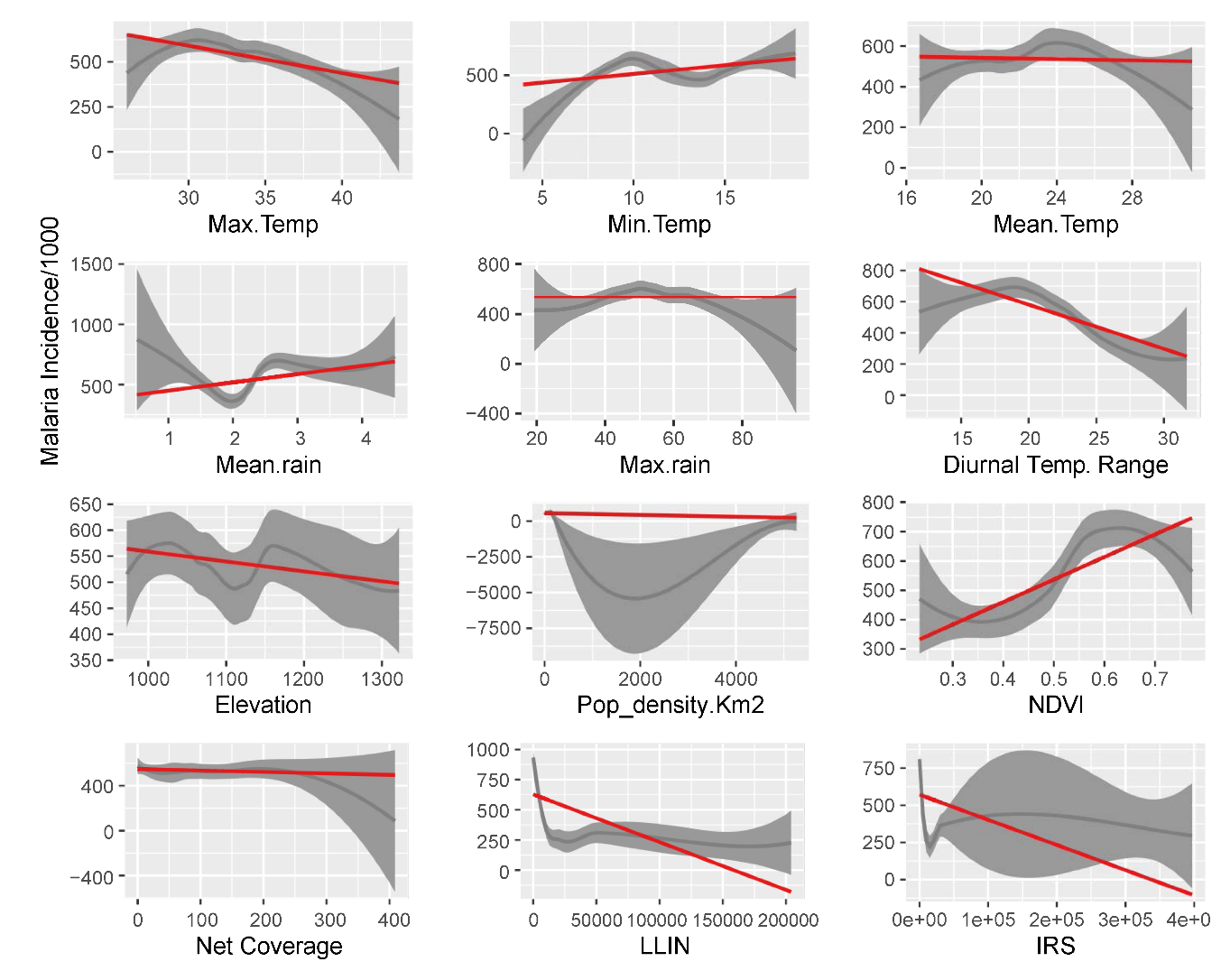


**Figure S3:** **Correlation of malaria incidence with key predictor variables in areas of declining malaria (using LOWESS smoothing function)*.*** Grey represents confidence intervals (95% CI) with 75% data. The red-line represents a linear fit of the distribution.


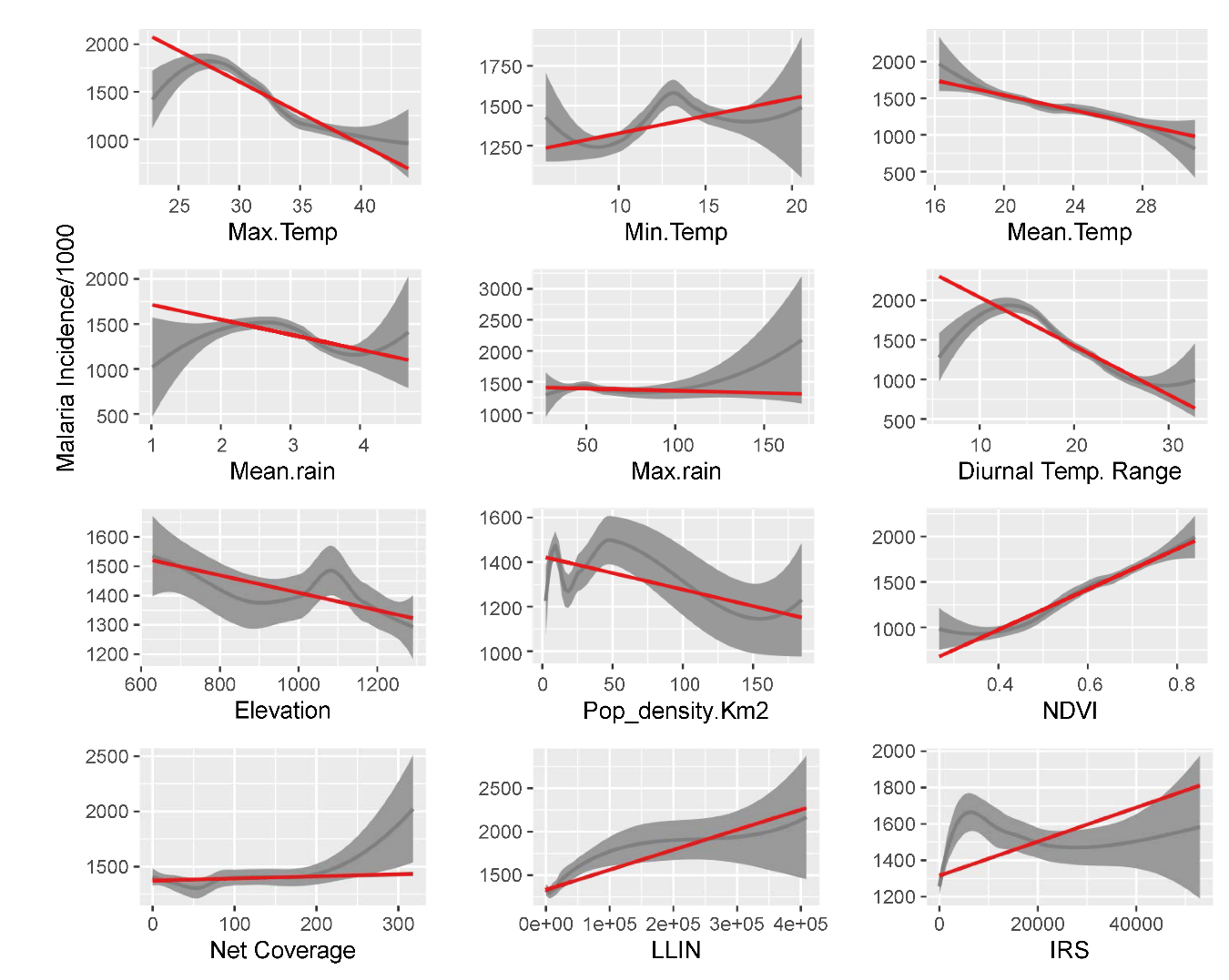


**Figure S4: Correlation of malaria incidence with key predictor variables in areas of increasing malaria (using LOWESS smoothing function)*.*** Grey represents confidence intervals (95% CI) with 75% data. The red-line represents a linear fit of the distribution

**
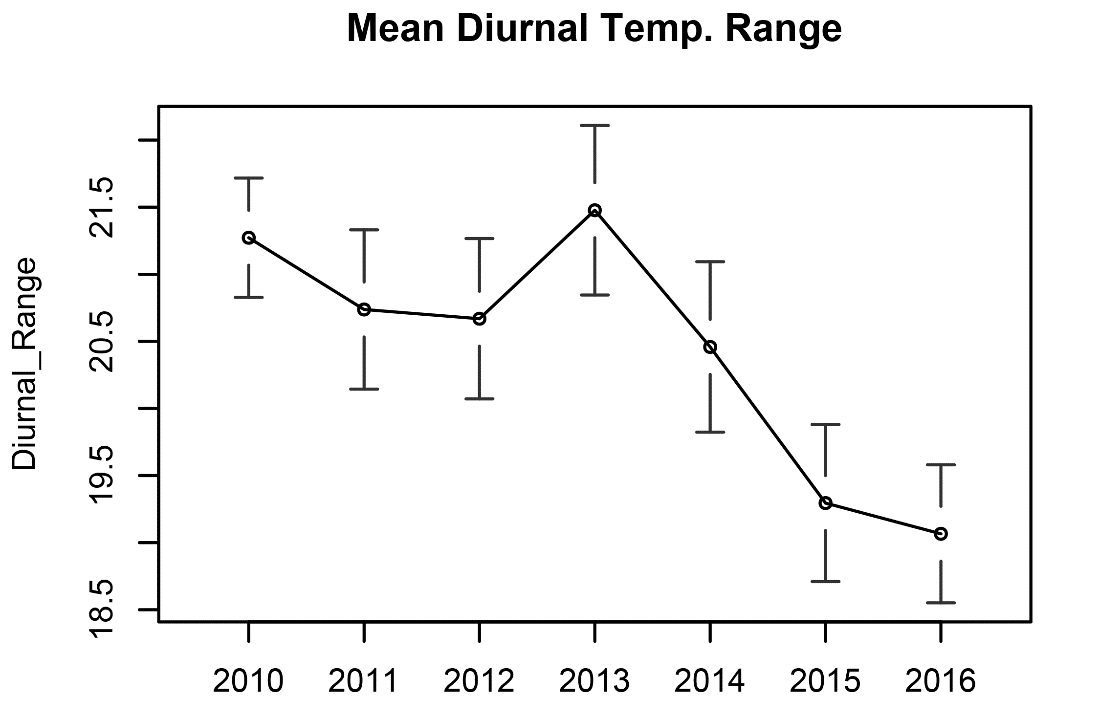
**

**Figure S5: Overall mean DTR trend from 2010-2016.**

The error bars represent (95% CI)

**Figure S6a: Areas of increasing malaria**


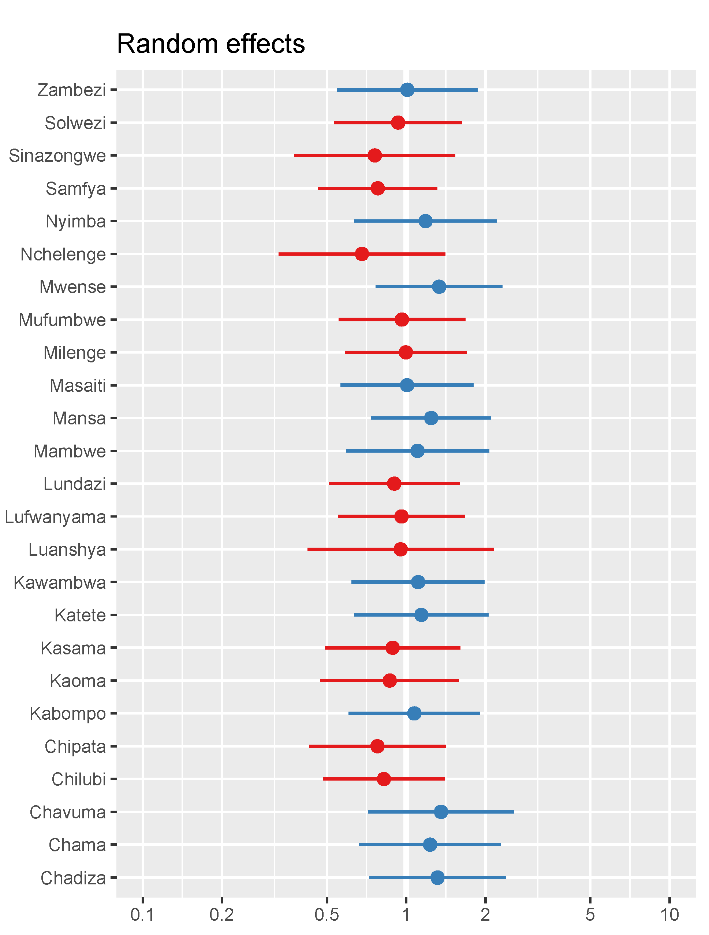

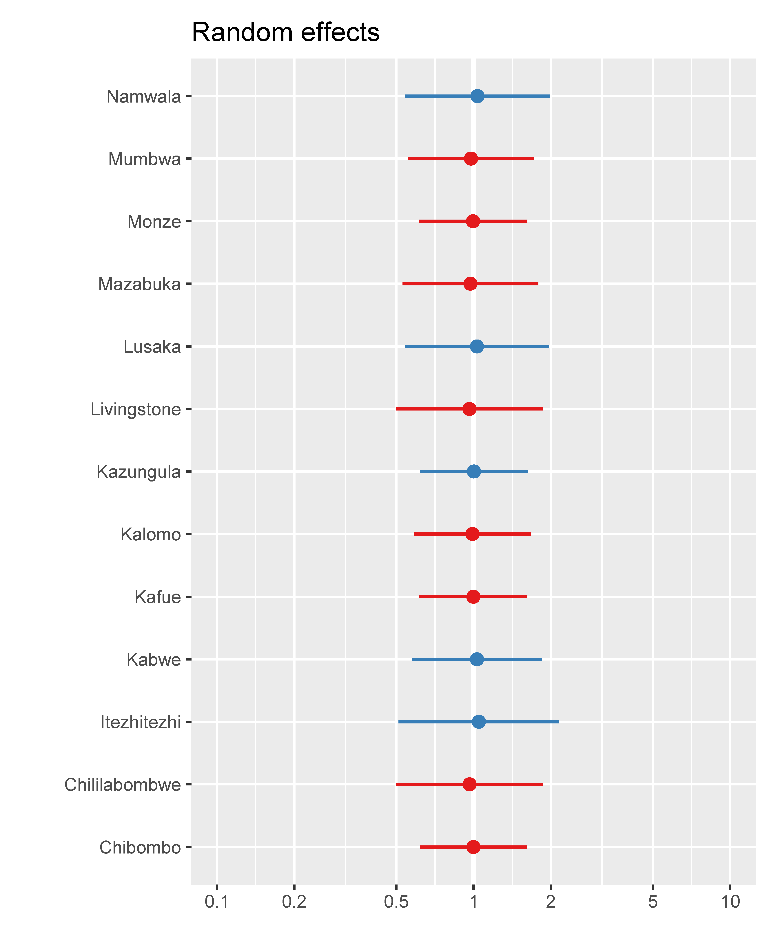


**Figure S6b: Areas of declining malaria**


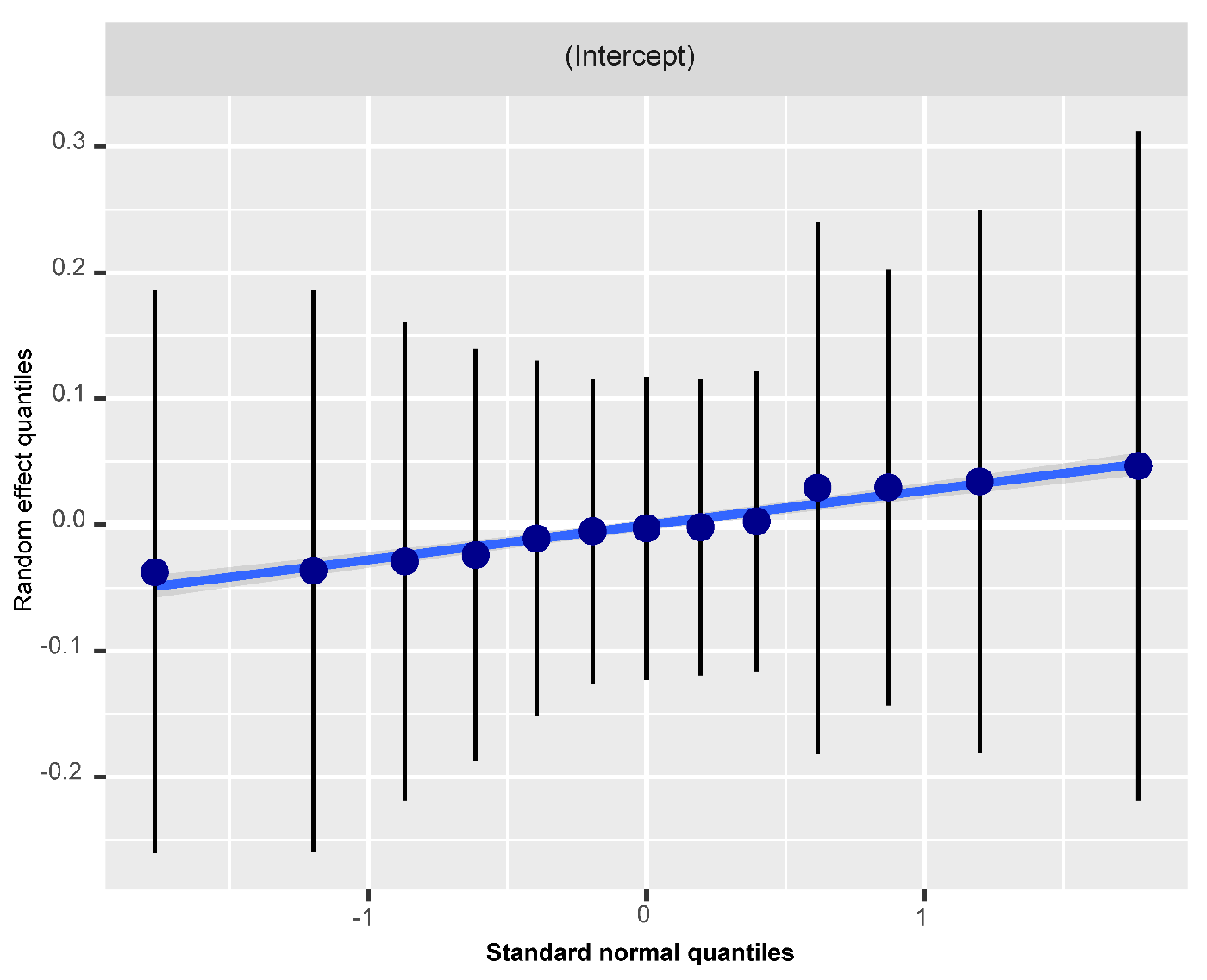


**Figure S7:** **Random effect quantiles in areas of declining malaria**


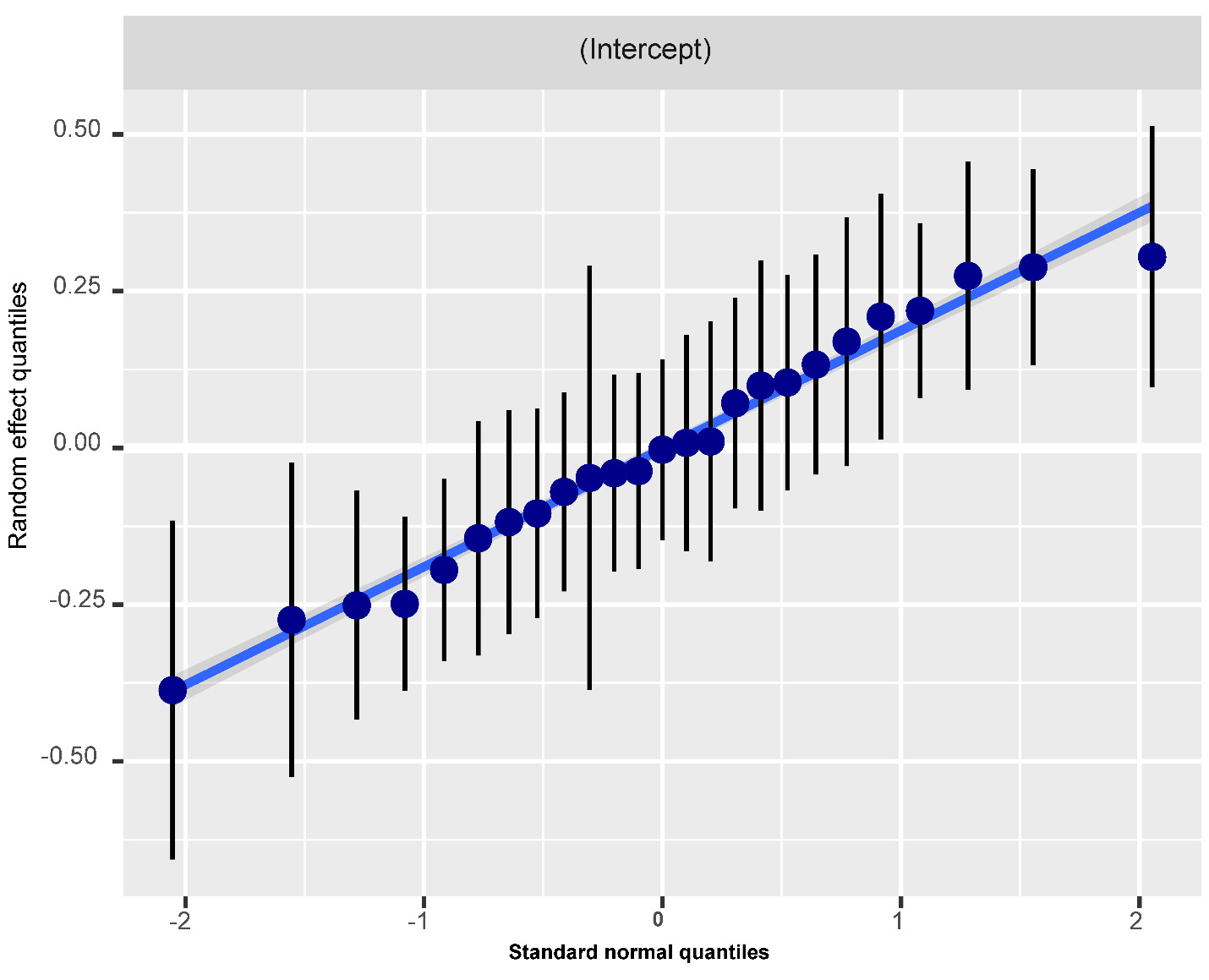


**Figure S8:** **Random effect quantiles in areas of increasing malaria**


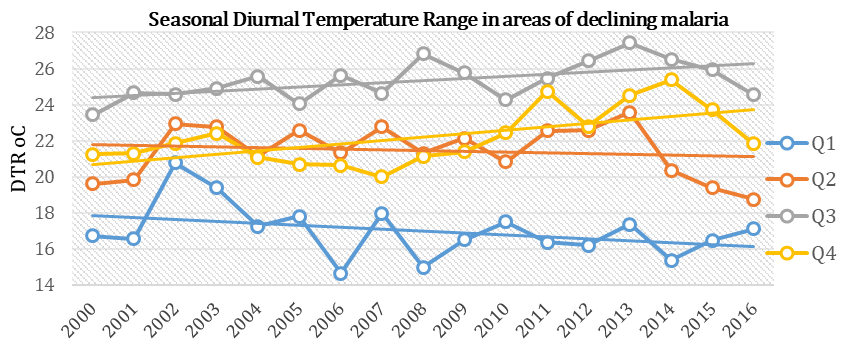

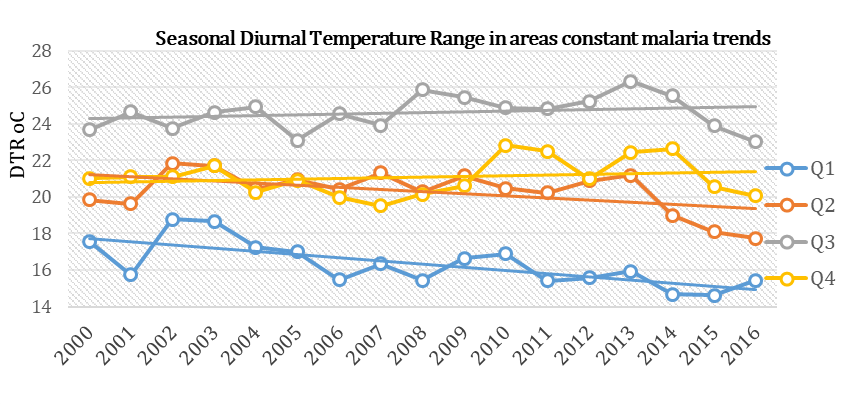

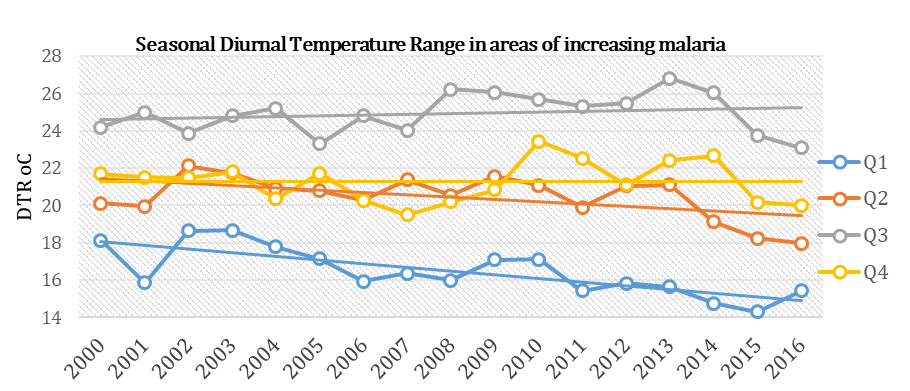


**A**

**Figure S9: Seasonality of Diurnal Temperature Range (DTR) in areas of decline, increase, or constant trends**

**Q1:** y = -0.1748x + 17.896; R² = 0.51. **Q2:** y = -0.1168x + 21.355; R² = 0.26

**Q3:** y = 0.0416x + 24.229; R² = 0.05. **Q4:** y = 0.0392x + 20.729; R² = 0.04

Q1: y = -0.1089x + 17.989; R² = 0.1345. Q2: y = -0.042x + 21.82; R² = 0.0215

Q3: y = 0.1174x + 24.277; R² = 0.303. Q4: y = 0.1897x + 20.481; R² = 0.3739

**Q1:** y = -0.1983x + 18.264; R² = 0.59. **Q2:** y = -0.1252x + 21.594; R² = 0.29

**Q3:** y = 0.0411x + 24.552; R² = 0.04. **Q4:** y = 0.0009x + 21.274; R² = 2E-05

**B**

**C**


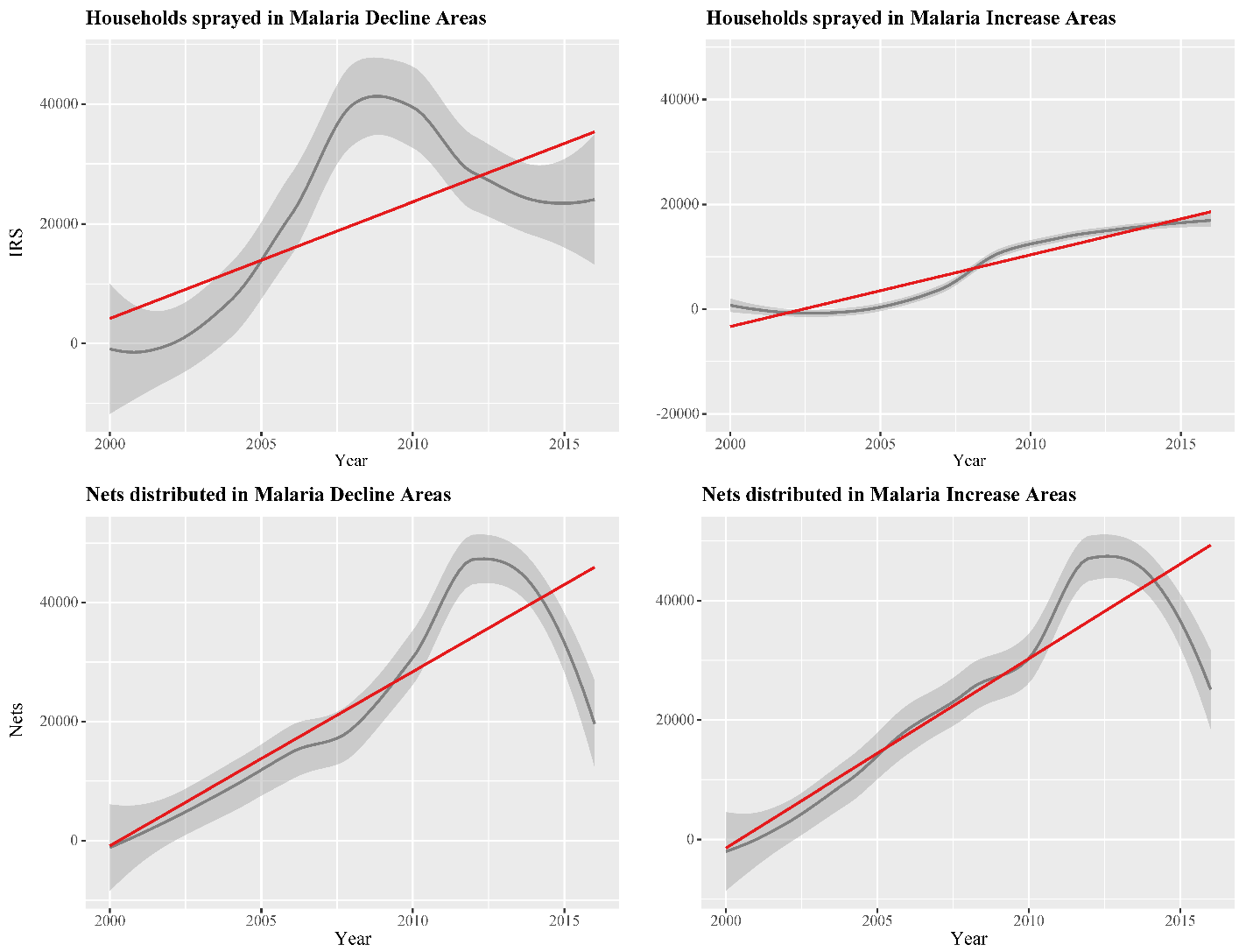


**Figure S10: Malaria interventions implemented in areas of malaria decline vs increase (using LOWESS smoothing function).**

Grey represents confidence intervals (95% CI) with 75% data. The red-line represents a linear fit of the distribution

**Figure S11: Spatial variation of malaria incidence from the year 2000**


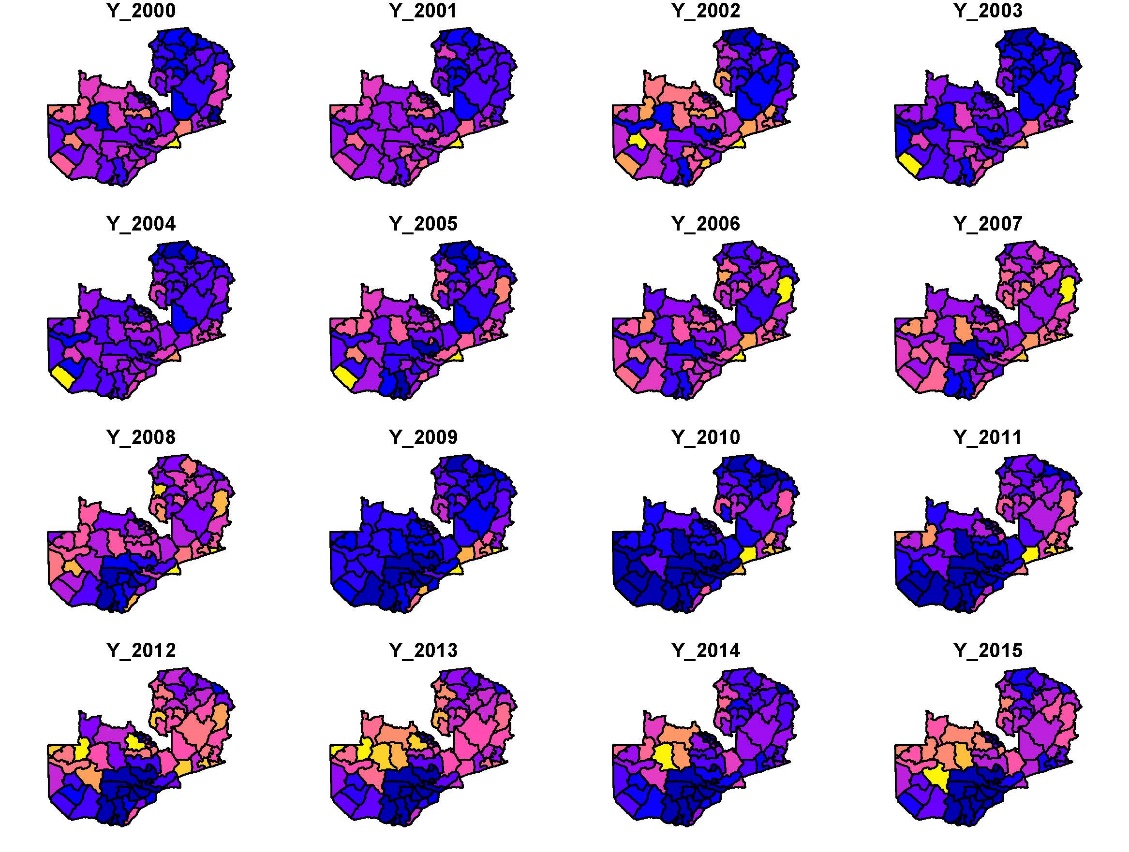

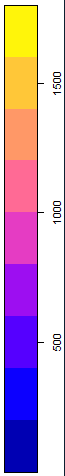


Figure S11 shows that shifts in malaria transmission incidence date earlier than 2008 but somewhat become more apparent then and the trend continues post-2008.

**Table S2: Summary statistics of environmental variables between the two groups**

|  | Declining malaria | | | Increasing malaria | | |
| --- | --- | --- | --- | --- | --- | --- |
|  | Mean | Min | Max | Mean | Min | Max |
| *Max Temp* | 33.4 | 26.0 | 43.7 | 33.5 | 22.9 | 43.9 |
| *Min Temp* | 11.9 | 4.0 | 18.9 | 12.7 | 5.8 | 19.5 |
| *Mean Temp* | 22.6 | 16.7 | 31.2 | 23.1 | 16.3 | 31.1 |
| *NDVI* | 0.5 | 0.2 | 0.8 | 0.6 | 0.3 | 0.8 |
| *Mean daily Rain* | 2.3 | 0.5 | 4.5 | 2.9 | 1.0 | 4.3 |
| *Max daily Rain* | 50.9 | 19.3 | 95.7 | 58.6 | 27.2 | 171.0 |
| *Diurnal Range* | 21.5 | 11.9 | 31.7 | 20.8 | 6.0 | 32.8 |
